# Inhibition of methyltransferase activity of enhancer of zeste 2 leads to enhanced lipid accumulation and altered chromatin status in zebrafish

**DOI:** 10.1186/s13072-020-0329-y

**Published:** 2020-02-12

**Authors:** Marjo J. den Broeder, Jarle Ballangby, Leonie M. Kamminga, Peter Aleström, Juliette Legler, Leif C. Lindeman, Jorke H. Kamstra

**Affiliations:** 1 Institute for Risk Assessment Sciences, Faculty of Veterinary Medicine, Utrecht University, Utrecht, The Netherlands; 2 Faculty of Veterinary Medicine, Norwegian University of Life Sciences, Oslo, Norway; 3 Department of Molecular Biology, Faculty of Science, Radboud University Nijmegen, Radboud Institute for Molecular Life Sciences, Nijmegen, The Netherlands; 4 Wageningen University and Research Library, Wageningen, The Netherlands; 5 Utrecht Institute for Pharmaceutical Sciences, Faculty of Science, Utrecht University, Utrecht, The Netherlands

**Keywords:** Zebrafish, Epigenetics, Histone methyl transferases, ATAC-seq, Metabolism

## Abstract

**Background:**

Recent studies indicate that exposure to environmental chemicals may increase susceptibility to developing metabolic diseases. This susceptibility may in part be caused by changes to the epigenetic landscape which consequently affect gene expression and lead to changes in lipid metabolism. The epigenetic modifier enhancer of zeste 2 (Ezh2) is a histone H3K27 methyltransferase implicated to play a role in lipid metabolism and adipogenesis. In this study, we used the zebrafish (*Danio rerio*) to investigate the role of Ezh2 on lipid metabolism and chromatin status following developmental exposure to the Ezh1/2 inhibitor PF-06726304 acetate. We used the environmental chemical tributyltin (TBT) as a positive control, as this chemical is known to act on lipid metabolism via EZH-mediated pathways in mammals.

**Results:**

Zebrafish embryos (0–5 days post-fertilization, dpf) exposed to non-toxic concentrations of PF-06726304 acetate (5 μM) and TBT (1 nM) exhibited increased lipid accumulation. Changes in chromatin were analyzed by the assay for transposase-accessible chromatin sequencing (ATAC-seq) at 50% epiboly (5.5 hpf). We observed 349 altered chromatin regions, predominantly located at H3K27me3 loci and mostly more open chromatin in the exposed samples. Genes associated to these loci were linked to metabolic pathways. In addition, a selection of genes involved in lipid homeostasis, adipogenesis and genes specifically targeted by PF-06726304 acetate via altered chromatin accessibility were differentially expressed after TBT and PF-06726304 acetate exposure at 5 dpf, but not at 50% epiboly stage. One gene, *cebpa,* did not show a change in chromatin, but did show a change in gene expression at 5 dpf. Interestingly, underlying H3K27me3 marks were significantly decreased at this locus at 50% epiboly.

**Conclusions:**

Here, we show for the first time the applicability of ATAC-seq as a tool to investigate toxicological responses in zebrafish. Our analysis indicates that Ezh2 inhibition leads to a partial primed state of chromatin linked to metabolic pathways which results in gene expression changes later in development, leading to enhanced lipid accumulation. Although ATAC-seq seems promising, our in-depth assessment of the *cebpa* locus indicates that we need to consider underlying epigenetic marks as well.

## Background

In the last decades, the incidence of metabolic diseases such as obesity, type 2 diabetes mellitus (T2DM) and non-alcoholic fatty liver disease (NAFLD) has increased enormously in adults, but also alarming increases of these diseases have been observed among children and young adults [1, 2]. Next to changes in lifestyle and genetic predisposition, recent studies have shown that environmental factors may also play an important role in the development of metabolic diseases, possibly via altered (epi)genetic status [3]. Exposure during early life stages to metabolism disrupting chemicals (MDCs) has been identified as an environmental factor which may play a role in the development of metabolic diseases later in life. MDCs are a class of endocrine disrupting chemicals that have the ability to promote metabolic changes that may lead to increased susceptibility to develop obesity, T2DM or fatty livers in animals and humans [4, 5].

Experimental evidence indicates that developmental exposures to MDCs can induce changes to epigenetic programming leading to altered metabolism and latent onset of metabolic diseases [3, 6]. Epigenetics describes processes that affect how DNA is wrapped into chromatin and expressed without altering its sequence, ultimately shaping a phenotype [7]. Several different types of epigenetic modifications are thought to play a role in regulating metabolism, including DNA methylation, histone post-translational modifications (PTMs), and non-coding RNA molecules, each influencing an open or a closed state of chromatin depending on the type and location of the modification [8]. As a consequence, a more open transcriptionally permissive (euchromatin) or a closed repressive chromatin (heterochromatin) structure leads to either more active or repressed genes [9]. Ablation of specific histone modifications can shift the homeostatic balance of PMTs resulting in a change in gene expression, and consequently leading to a distinct phenotype such as altered lipid metabolism.

One of the factors that could play an important role in this process is enhancer of zeste 2 (EZH2), the catalytic factor of the Polycomb Repressive Complex 2 (PRC2) that places a methylation mark on histone H3 lysine 27 (H3K27me1, 2 or 3) through its methyltransferase activity [10]. For instance, in mice and rodent cell lines it has been shown that repression of Wnt genes through H3K27 methylation by EZH2 in preadipocytes is required for in vitro adipocyte differentiation [11]. In contrast, inhibition of EZH2 showed enhanced lipid accumulation in breast cancer cell lines and in a hepatocyte (HepG2) cell line [12, 13].

Enhanced adipogenesis as an adverse effect of exposure to MDCs is often accompanied by changes in epigenetic gene regulation as previously found [14–20]. For example, prenatal exposure to the MDC tributyltin (TBT) increases the number of adipocytes in mice offspring [21]. Additionally, prenatal exposure to TBT leads to a change in adipogenesis in the F4 generation, which is linked to changes in sperm chromatin structures. Of importance, activation of the retinoid X receptor (RXR) by TBT or a RXR-selective agonist (IRX4204) showed a reduced expression of EZH2 [20]. As a consequence, redistribution and an overall decrease of H3K27me3 were observed, especially close to genes involved in adipogenesis [20]. From the above it becomes evident that histone PTMs via EZH2 play a role in adipogenesis, but other PTMs or DNA methylation might play an equal important role in shaping the epigenetic landscape. Also, exposure to TBT in zebrafish leads to enhanced lipid accumulation [22] and adipogenesis in zebrafish embryos and larvae at low concentration [23]. Therefore, we hypothesize that TBT can act through Rxr and down-regulate the expression of *ezh2*. Next to that, we hypothesize that inhibition of Ezh2 protein in zebrafish lead to altered lipid accumulation as an effect of a more open chromatin status and an accompanied change in gene expression.

In this study, we use zebrafish (*Danio rerio*) as a model to investigate inhibition of Ezh2 on lipid metabolism. Transparent zebrafish embryos allow the visualization of developmental phenotypes following exposure to chemicals. Also, many protein coding genes in the zebrafish genome are conserved, including *ezh1* and *ezh2* and other Polycomb genes, showing high similarity with higher vertebrates as humans [24]. However, only Ezh2 is required for zebrafish embryo development. Although the role of Ezh2 during early development is studied quite in depth, the role of Ezh2 in lipid metabolism in zebrafish is unknown. We investigated the effect of inhibition of the histone methyl transferase (HMT) activity of enhancer of zeste proteins on zebrafish development with focus on lipid accumulation, and chromatin accessibility due to reduced H3K27me3 levels. Therefore, we exposed zebrafish embryos to the Ezh inhibitor PF-06726304 acetate [25], and measured changes on chromatin structure by the assay for transposase-accessible chromatin sequencing (ATAC-seq), just after zygotic genome activation (ZGA) at 50% epiboly.

The zebrafish has become an important model in toxicology to study the effects of chemicals on early development and adults, and is used in transgenerational studies as well. However, most research focus on phenotypic effects, often accompanied with changes in gene expression, but underlying epigenetic mechanisms are less prominently studied. In zebrafish, the chromatin becomes remodeled during mid-blastula transition (MBT) and zygotic genome activation (ZGA) accompanied with changes in histone methylation [26–28], however knowledge is lacking about the effect of exposures to chemicals on histone modifications and chromatin status. Most environmental epigenetic research has focused at DNA methylation or non-coding small RNA molecules [29], but emerging studies investigate the role of higher order chromatin structures via post-translational histone modifications [20, 30, 31] and chromatin structural analyses [32]. Here, we investigated if chromatin accessibility can be used as an end-point in toxicological research as an indicator for change in gene expression directly after exposure and later in life.

In this study, we show that exposure to the Ezh2 inhibitor, PF-06726304 acetate, has impact on lipid accumulation in 5-dpf-old larvae. Importantly, we detected changes in chromatin status linked to H3K27me3 loci shortly after genome activation, which could be further linked to biological processes involved in metabolism. Furthermore, we characterized specific differentially regulated genes in order to gain knowledge on the specific mode of action of PF-06726304 acetate.

## Results

### Developmental exposure to PF-06726304 acetate leads to moderate teratogenicity

**Fig. 1:**
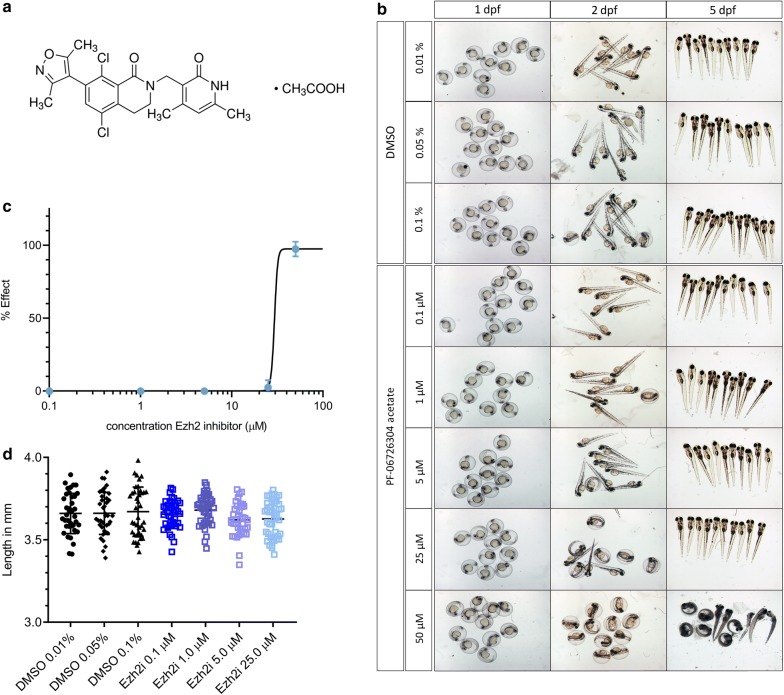
Phenotypic effects of PF-06726304 acetate (Ezh2i). **a** Structural formula of PF-06726304 acetate (source: http://www.sigmaaldrich.com). **b** Developmental exposure to a concentration range of Ezh2i at 1, 2 and 5 days post-fertilization (dpf). **c** Concentration–response curve of Ezh2i at 5 dpf. **d** Length measurements of larvae following exposure to Ezh2i at 5 dpf. Error bars indicate standard deviation (SD)

To test whether inhibition of Ezh2 proteins may lead to a phenotypic effect on zebrafish embryonic development, we exposed embryos from 0 to 5 days post-fertilization to different concentrations of the Ezh2 inhibitor PF-06726304 acetate (Fig. 1a; referred to as Ezh2i) (0.1 μM, 1 μM, 5 μM, 25 μM and 50 μM) and corresponding solvent controls (0.01, 0.05 an 0.1% DMSO) (Fig. 1b, c).

At 2 dpf, all the embryos within control and tested concentrations up to 25 μM Ezh2i had well-defined pigmented eyes, heartbeat and developed normally. The first signs of abnormalities such as heart sac edema, were observed in > 50% of embryos exposed to 50 μM of Ezh2i. All the embryos with heart edema had impaired blood circulation, as blood accumulation was visible under the yolk sac and around the heart. At 5 dpf, all embryos in controls and tested concentrations up to 25 μM were hatched and continued to grow, however, larvae exposed to 50 μM of Ezh2i showed a lethal effect to the compound in all replicates. At this concentration, all the uncoagulated embryos failed to progress through development, some with clear body plan and eyes developed, but failed to hatch and died. A concentration–response curve of Ezh2i revealed an EC50 of 29 μM (Fig. 1c).

To further investigate the possible effects on general development and health of larvae, we measured the standard length (SL) of 5-dpf-old larvae. The average SL of larvae in control group was 3.7 mm. Overall, the average size of the larvae measured in each tested group lies between 3.6 and 3.8 mm. No significant difference in size was measured when comparing solvent control and tested concentrations of Ezh2i (Fig. 1d).

### Exposure to Ezh inhibitor PF-06726304 acetate augments lipid accumulation

**Fig. 2:**
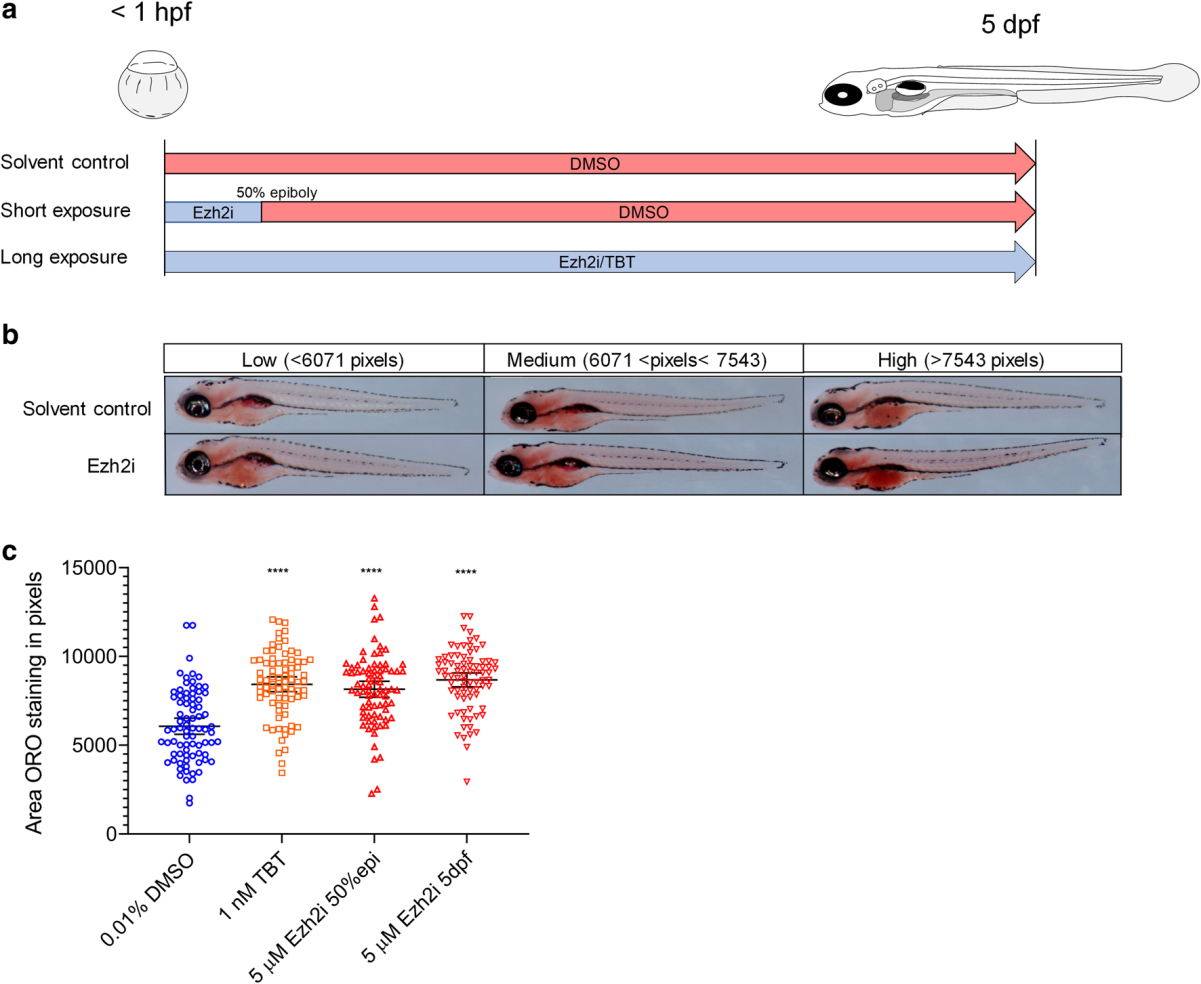
Lipid accumulation after exposure to PF-06726304 acetate (Ezh2i) and tributyltin (TBT). **a** Experimental design. Embryos are either exposed to Ezh2i or TBT in a short or long exposure window. **b** Representative images for ORO staining with low, medium or high staining levels. **c** Scatter plot of ORO staining following the different treatments. Average and error bars (95% CI) indicated in black (****padj-value < 0.0001)

From our concentration–response curve analysis, we chose a non-toxic concentration of 5 μM for all our subsequent analyses. We performed a short (0 to 50% epiboly exposure, recovery up to 5 dpf; referred as Ezh2i50%epi samples) and long window exposure to Ezh2i (0–5 days exposure; Fig. 2a). A positive control (1 nM TBT) and a negative control (0.01% DMSO) were included in the set-up (Fig. 2a). We performed neutral lipid dye Oil Red O (ORO) staining on paraformaldehyde fixed larvae, and these stained larvae showed presence of lipid staining in brain and abdominal area at different intensities at 5 dpf (Fig. 2b). To quantify the lipid staining, we measured the number of red pixels of the yolk sac area in control and exposed 5-dpf-old larvae using ImageJ. The experiment was performed in duplicate, and a two-way ANOVA analysis showed that there were no significant differences between the two independent experiments. Accumulated data from the two experiments showed significantly increased lipid accumulation compared to solvent control in larvae exposed to the positive control TBT (1 nM), and the short and the long Ezh2i exposure (Fig. 2c). The mean value of solvent control group was 6071 pixels, compared to 7543 pixels for TBT, 7584 pixels Ezh2i50%epi and 8680 pixels Ezh2i5dpf samples.

### ATAC-seq reveals altered chromatin accessibility at H3K27me3 loci

**Fig. 3:**
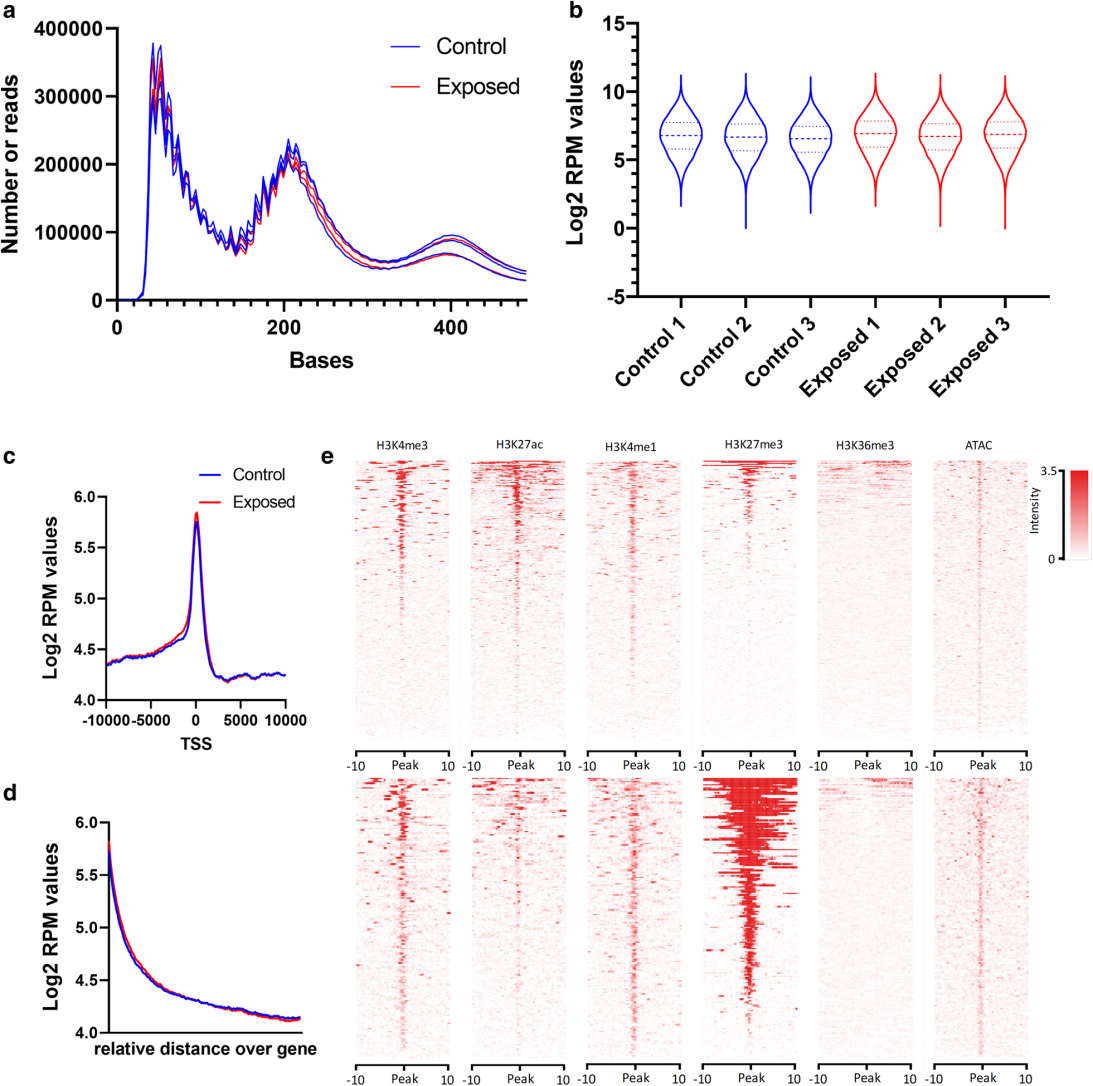
ATAC sequencing results of 50% epiboly embryos exposed to PF-06726304 acetate. **a** Read length distribution of sequences showing a typical ATAC fingerprint. **b** Violin plot showing log2 read per million (RPM) values of all ATAC peaks (dashed lines indicate median and quartiles). **c** Enrichment plot showing the global mapping of sequencing reads mapped around the transcriptional start sites (TSSs) of all genes. **d** Enrichment plot showing the global mapping of sequencing reads mapped over the relative length of all genes. **e** Density plots of different histone marks and accessible chromatin at dome stage around (upper part) all ATAC peaks and (lower part) differential enriched ATAC peaks located at TSSs spanning a region of 10 kb

Sequences generated from the ATAC samples were of high quality with phred scores > 30 for all samples (Additional file 1: Fig. S1). The mapping to the GRCz11 zebrafish genome resulted in around 40 million unique alignments on average per sample (Additional file 1: Fig. S2). The alignments showed a typical ATAC profile, identifying nucleosome positions around 200 and 400 bp (Fig. 3a) [33]. In total 65336 peaks were identified using MACS2. Generally, the Log2 reads per million values of the identified peaks showed a marginal increase towards the Ehz2 inhibitor (Fig. 3b). From the 65,336 peaks, 22,026 were overlapping with genic regions (defined as 2000 bases ± mRNA region), and 19,331 peaks were directly located at transcriptional start sites (TSS) (defined as 1000 bases ± TSS of mRNAs). A typical enrichment of ATAC peaks at TSSs and a decline of enrichment over gene bodies are shown in Fig. 3c, d with no apparent difference between exposures. From the peaks located at TSSs, 349 were identified as differentially enriched (DE) between the Ezh2 inhibitor and controls. When comparing the data with previous published data on H3K4me3, H3K27ac, H3K4me1, H3K27me3, H3K36me3 and ATAC from dome stage embryos [34–36], our data follow expected patterns resulting from Ezh2 inhibition. Enrichment plots of the 19331 ATAC peaks located at TSSs as shown in the upper panel of Fig. 3e show typical enrichments that are expected from ATAC data at H3K4me3/1, H3K27ac/me3 locations, but none at H3K36me3. A very sharp enrichment pattern is observed with previously published ATAC data, indicating that the locations of the peaks in this study are highly alike with the previous reported data. When focusing on the 349 DE peaks, a significant enrichment at H3K27me3 locations is observed, indicating that indeed H3K27me3 is targeted by the Ezh2i (Fig. 3e lower panel).

### Differential ATAC peaks are linked to pancreatic cell development and metabolic pathways

**Fig. 4:**
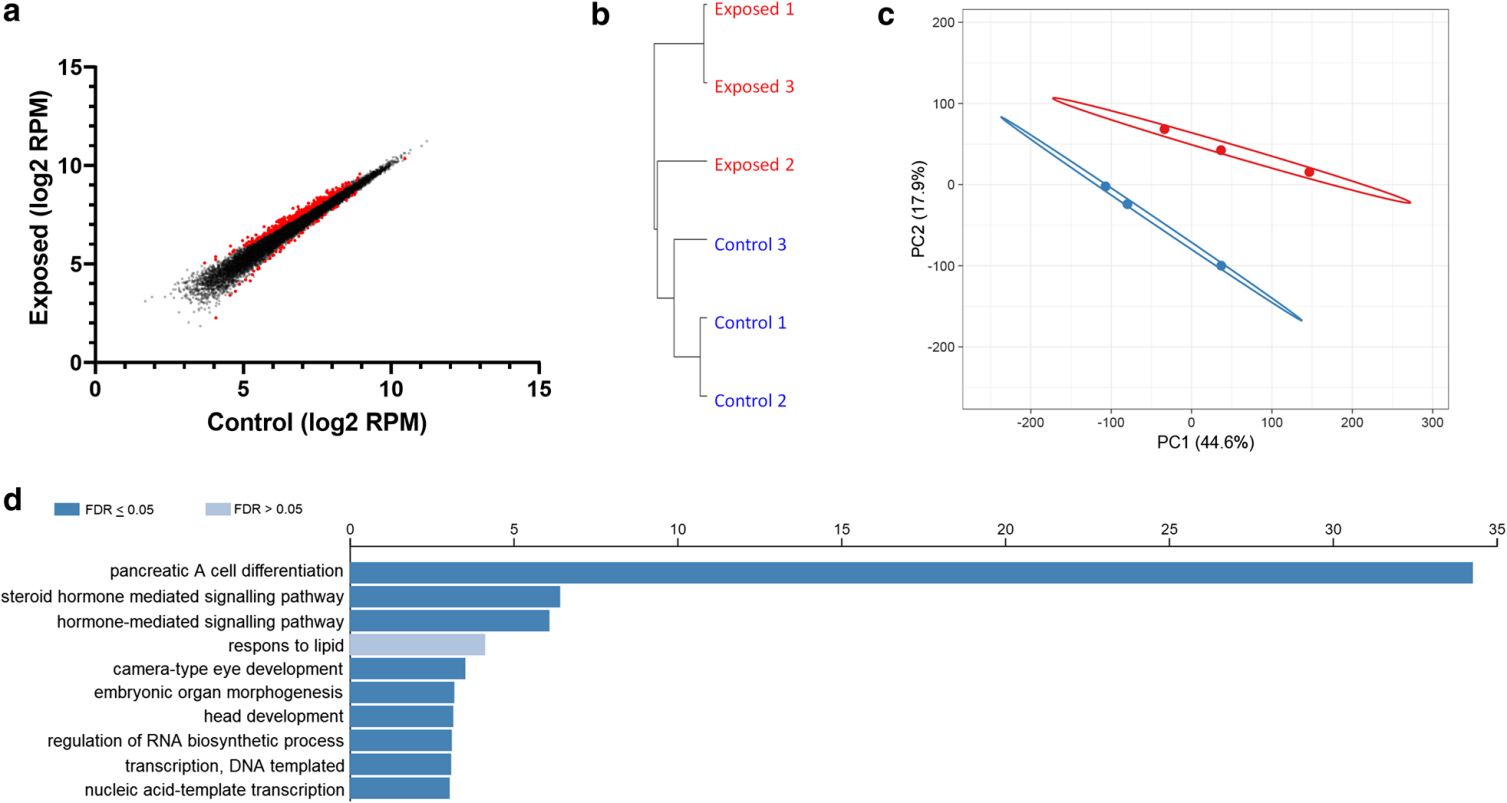
Differential enrichment (DE) analysis of ATAC peaks at transcriptional start sites (TSSs). **a** Scatterplot of peaks located at TSSs. Red dots indicate differentially enriched ATAC peaks. **b** Cluster analysis (Pearson correlation). **c** Principal component analysis of ATAC peaks located at TSSs (control in blue, exposed in red). **d** Top 10 gene ontology enrichment analysis (GO term biological process) of DE peaks at TSSs

From the 349 DE peaks, 302 were increased, representing more open chromatin, as is expected from H3K27me3 inhibition (Fig. 4a). Correlation analysis showed a clear clustering of all exposed Ezh2i samples vs controls (Fig. 4b). Additionally, principal component analysis (PCA) revealed a separation between the controls and exposed samples. Principal component 1 (PC1) and PC2 explained 44.6% and 17.2% of the total variance between the samples (Fig. 4c). Gene Ontology analysis showed most biological processes are enriched for processes linked to pancreatic alpha cell differentiation, hormone mediated signaling and response to lipids (Fig. 4d). Many metabolic processes were affected as is shown in the GO list per term in Additional file 2.

### Differential ATAC peaks link to differential expressed genes from MZehz2 mutants

**Fig. 5:**
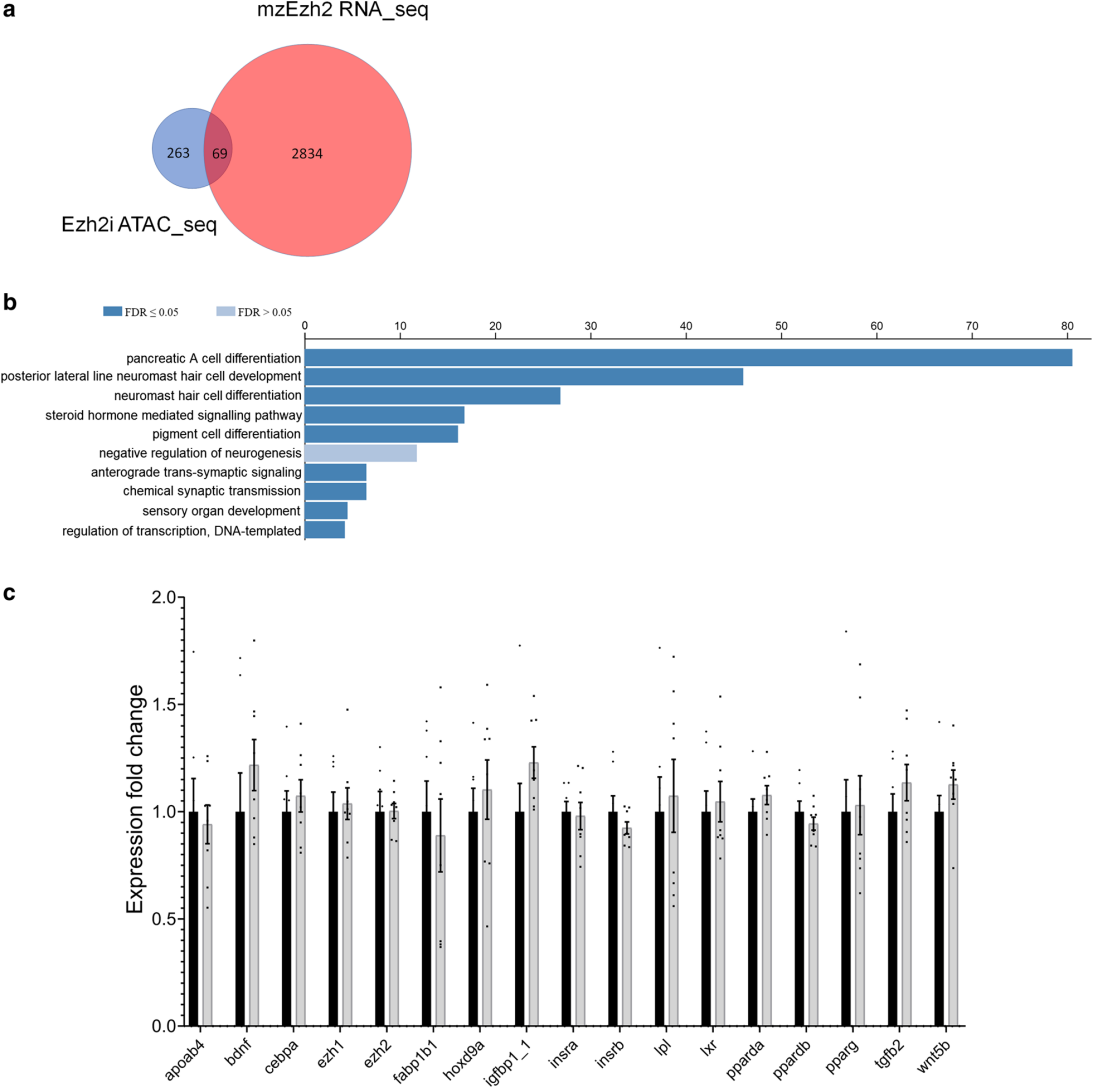
Gene expression at 50% epiboly is not significantly changed. **a** Venn diagram showed an overlap of 69 genes between DEP from ATAC sequencing and DEG after RNAseq on *MZezh2* embryos. **b** Top 10 gene ontology enrichment analysis (GO term biological process) for 69 overlapping genes between ATAC-seq and *MZezh2* RNA-seq. **c** Gene expression at 50% epiboly shown with standard error of mean (SEM)

To test which chromatin regions are specifically targeted by the inhibitor, we compared a differentially expressed gene (DEG) list (padj < 0.05; 2904 genes) of *MZehz2* mutants with our DE peaks gene list [37]. *MZezh2* embryos do not possess the H3K27me3 mark on the genome and gene expression data should directly link to our ATAC data set. Using a Venn diagram we observed an overlap of 69 genes that were shared in both datasets (Fig. 5a), which is more than expected based on random sampling (*P* < 0.0001, Fisher exact test). Interestingly, gene ontology analysis of these 69 genes showed again an enrichment for pancreatic A cell differentiation (Fig. 5b), but also other processes in metabolism and lipid homeostasis (Additional file 2).

### Gene expression at 50% epiboly is not significantly changed

Embryos exposed to 5 μM Ezh2i or solvent control (0.1% DMSO) at 50% epiboly were analyzed for gene expression of a selection of genes from the overlapping ATAC-seq and RNA-seq gene list. In addition, we analyzed gene expression of adipogenic genes expressed as early as 50% epiboly. No effects of Ezh2 chemical inhibition was found on expression of these genes at the 50% epiboly stage (Fig. 5c).

### Long exposure to Ezh2i and TBT shows high similarity at gene expression level

**Fig. 6:**
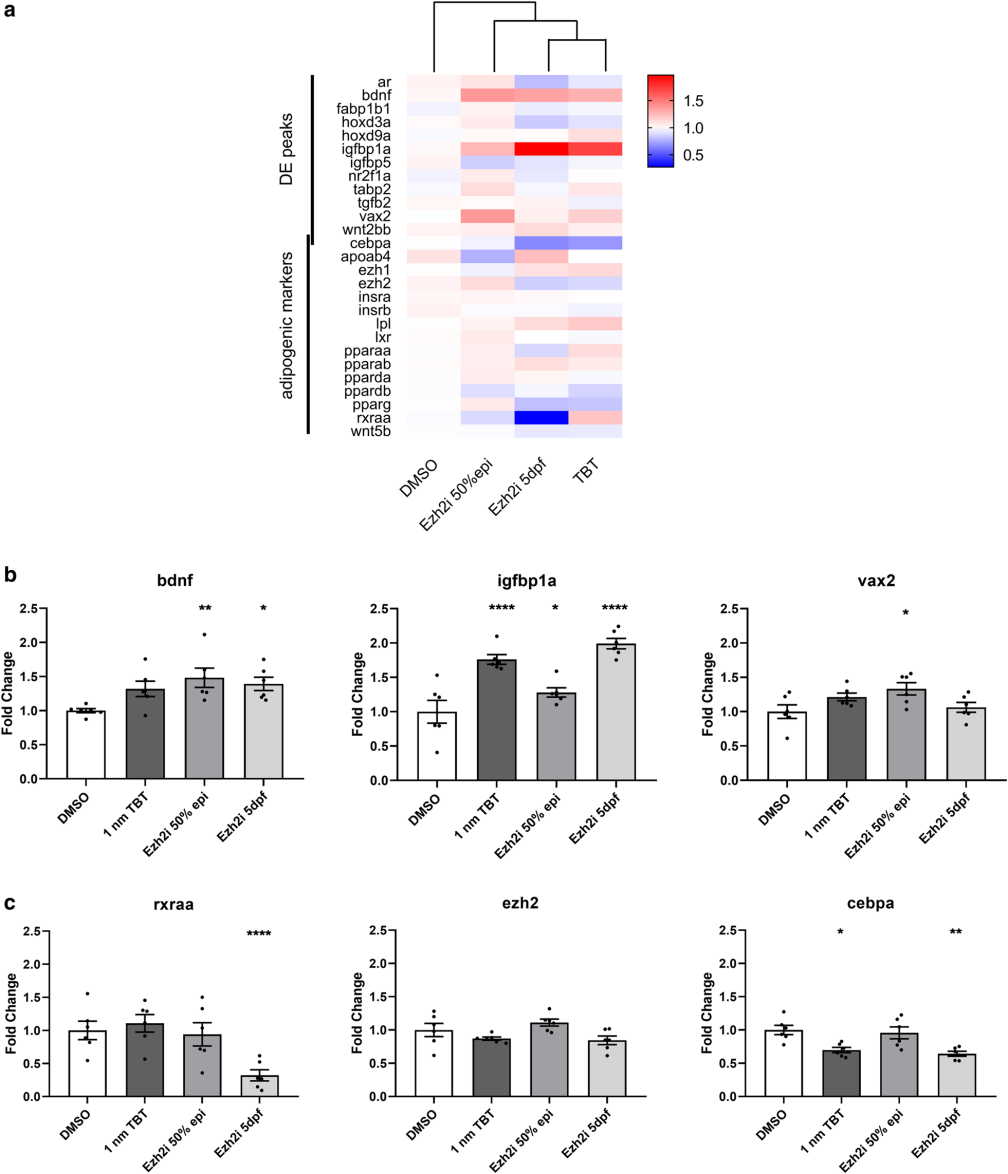
Gene expression analysis of genes involved in lipid processes or differential ATAC peaks. **a** Hierarchical clustering of gene expression. **b** Upregulated genes that also showed differential enhanced peaks (more reads). **c** Down-regulated genes after exposure to Ezh2i (***padj-value < 0.0001; ***padj-value < 0.001; **padj-value < 0.01; *padj-value < 0.05). Error bar indicate standard error of mean (SEM)

Differences in gene expression between the different experimental groups were investigated using an overall comparison which is visualized in a heatmap by hierarchical clustering of experimental groups. Gene expression of TBT and Ezh2i5dpf samples show higher similarity compared to short exposure Ezh2i50%epiboly samples and solvent control group (Fig. 6a). *Brain derived neurotrophic factor (bdnf), insulin growth factor binding protein 1a (igfbp1a)* and *ventral anterior homeobox 2* (*vax2*) that had increased DE peaks after ATAC-seq also showed higher fold change expression with qPCR. *Bdnf* was significantly higher expressed in samples exposed either short or longer to Ezh2i, while *igfbp1a* is significantly higher expressed in TBT, Ezh2i5dpf samples and Ezh2i50%epiboly samples than solvent control samples. *Vax2* was only significantly higher expressed in short exposure Ezh2i50%epiboly samples (Fig. 6b). Interestingly, the *retinoid X receptor alpha a* (*rxraa*) is strongly down-regulated in the Ezh2i5dpf samples, without any change in *enhancer of zeste 2* (*ezh2*) gene expression. *CCAAT enhancer*-*binding protein alpha* (*cebpa)* showed significant reduced expression in TBT and Ezh2i5dpf samples (Fig. 6c).

**Fig. 7:**
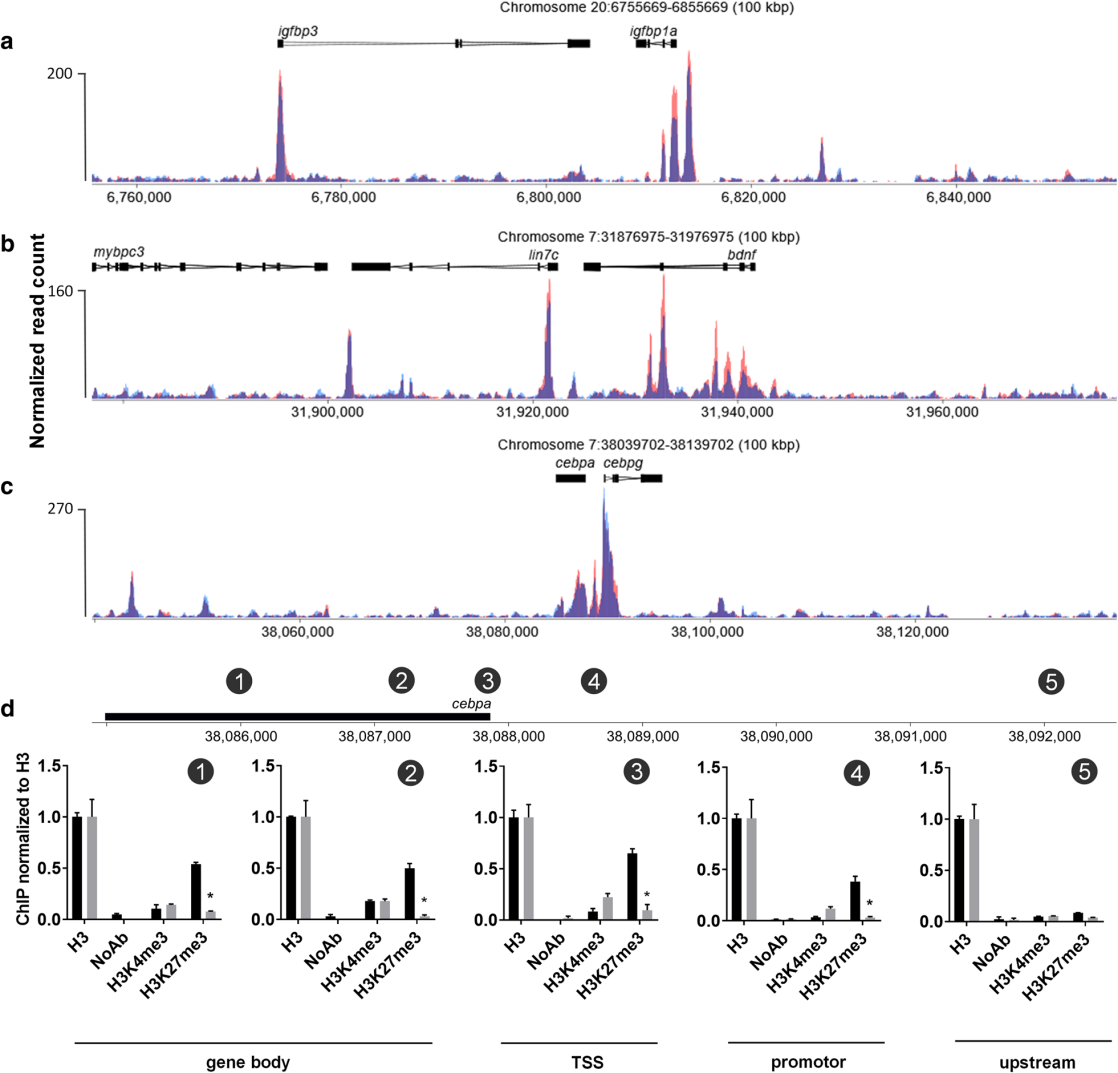
Differential expressed genes versus ATAC results. Peak patterns around, **a** insulin growth factor binding protein 1a *(igfbp1a)*, **b** brain derived neurotrophic factor (*bdnf*) and **c** CCAAT/enhancer-binding protein alpha *(cebpa)* (blue is control and red is exposed). **d** Chromatin immunoprecipitation qPCR at 5 locations around the *cebpa* locus with histone H3 lysine 4 trimethylation (H3K4me3) and histone H3 lysine 27 trimethylation (H3K27me3) normalized against histone 3 (H3). Error bar reflects SEM. NoAb are no antibody controls. Black = control; grey = exposed. TSS: transcriptional start site

To further investigate the differences observed in gene expression after 5 days of exposure to Ezh2i, we related these changes back to the chromatin accessibility data. Both *bdnf* and *igfbp1a* showed a significant increase in enrichment at 50% epiboly (Fig. 7a, b). Although the gene expression did not change at 50% epiboly, the expression did increase at 5 dpf. On the other hand, at *cebpa* no changes were observed in chromatin accessibility (Fig. 7c), but a strong decrease in gene expression was observed at 5 dpf. To investigate this further we performed ChIP-qPCR of H3K27me3 at different locations of the *cebpa* locus and found significant demethylation of underlying H3K27me3 at all locations around the gene, but not further upstream (Fig. 7d).

## Discussion

Reprogramming of the epigenome is thought to be one of the mechanisms by which early developmental exposure can influence disease susceptibility later in life, and possibly be transmitted to next generations [38]. Here, we show that developmental exposure to Ezh2i increases lipid accumulation in developed zebrafish larvae, linked to changes to the epigenome and gene expression. Our results also indicate a primed state of chromatin in early development leading to changes later in life, indeed following the hypothesis of the developmental origins of health and disease [38].

The aim of this study was to investigate the role of Ezh2 inhibition on zebrafish embryo development and lipid accumulation in 5-dpf larvae, by using the newly developed Ezh inhibitor PF-06726304 acetate, targeting both Ezh1 and 2. However, this molecule will mainly inhibit the function of Ezh2 as Ezh1 is not expressed at detectable levels in zebrafish at early stages (up to 24 hpf), and remains low expressed until 6 dpf [39]. Furthermore, it has been shown that the PRC2–Ezh2 complex has higher HMT activity compared to the PRC2-Ezh1 complex [40].

Embryos treated with different concentrations of Ezh2i showed normal early development. However, we observed cardiac edema in some of the embryos exposed to Ezh2i but only at the highest tested concentration (50 µM) at 2 dpf, and mortality with an EC50 value of 29 µM at 5 dpf. Cardiac edema was also observed in embryos exposed to another Ezh inhibitor, DZNep [41], and myocardial defects are described in maternal zygotic mutant *MZezh2*^*hu5760/hu5760*^ embryos [42]. Zygotic *ezh2* mutants form a normal body plan, but die around 12 dpf [16, 17]. *MZezh2* embryos have normal gastrulation, develop a normal body plan but die at 2 dpf showing pleiotropic phenotypes due to loss of tissue maintenance [37, 42]. Actually, these *MZezh2* mutant embryos do not possess the H3K27me3 mark on the epigenome and gene expression analysis showed that genes important for early development are not switched off [37]. Although Ezh2 is essential for embryonic development and tissue maintenance in zebrafish, maternal load of Ezh2 mRNA is probably sufficient to add H3K27me3 mark to the chromatin in embryos as described previously in zygotic *ezh2* mutants [37, 42]. Ezh2i will block the SET domain of specifically Ezh1/2 proteins and thereby inhibit the catalytic function to add the H3K27me3 mark on the chromatin [43]. Indeed, when we map the ATAC DE peaks to H3K27me3 loci, we see a substantial over-representation at these locations. Reduced levels of H3K27me3 can lead to a more open chromatin status leading to higher expression of genes that normally are repressed. This can interfere with important biological processes during zebrafish development leading to non-viable larvae as seen in the Ezh2 mutant experiments.

By blocking the HMT activity of Ezh1/2 proteins by exposure to its inhibitor, we wanted to investigate the effect on lipid accumulation at a non-toxic concentration (5 µM). Lipid accumulation is enhanced both after short and longer exposure to Ezh2i as was shown by neutral lipid staining with Oil Red O, and at comparable levels as seen with positive control TBT. These findings corroborate with our earlier study with developmental exposure of TBT in zebrafish in which we observed a higher prevalence of larvae with adipocytes combined with higher amount of adipocytes in these larvae [23]. Additionally, other studies also found increased adiposity in either zebrafish larvae or juveniles [22, 44]. Enhanced adipogenesis may lead in turn to obesogenic phenotypes later in life, and related metabolic disorders [45]. Additionally, similar effects of enhanced lipid accumulation have been observed in human cell lines. For instance, breast cancer cell lines treated with Ezh2 inhibitors, DNZep and GSK126, showed more lipid droplets [12]. Also HepG2 cells treated with DNZep showed enhanced lipid accumulation [13].

To get a better understanding of possible mechanisms involved in the increased lipid accumulation, we performed ATAC sequencing just after ZGA, and observed 349 DE peaks directly located at TSS. As a consequence of altered H3K27me3 levels on the chromatin, most DE peaks had more reads compared to controls reflecting a more open chromatin state. Gene Ontology analysis showed enrichment of genes involved in pancreatic A cell differentiation, (steroid) hormone mediated signaling, important developmental genes, and neurological pathways. The variety in pathways and processes that have been altered is probably a result of the stochastic effect of Ezh2 protein inhibition as normally PRC2–Ezh2 complexes are recruited to multiple positions on the chromatin. Nevertheless, the over-representation of genes involved in metabolic processes could possibly be involved in the increased lipid accumulation observed in the embryos. Additionally, we compared our data with published gene expression data from *MZezh2* mutants and searched for specifically targeted chromatin regions by the inhibitor. Although the published RNA-seq data was performed at the earlier dome stage, the moment that the zygotic genome becomes activated, a large overlap in genes was observed, which was accompanied by a large overlap in pathways, again linked to metabolism.

Interestingly, an assessment of gene expression of a selection of these genes did not reveal changes in gene expression at 50% epiboly directly after Ezh2i exposure. The concentration of Ezh2i (5 µM) used for genetic and phenotypic analysis is well below the EC50 level, which might explain the lack of changes in gene expression. However, we observed mostly more open chromatin which indicates a change in nucleosome positioning favoring recruitment of transcription factors and RNA polymerase II leading to altered, presumable higher, gene expression. As we performed ATAC seq at such an early developmental stage (50% epiboly), specific required transcription factors might not be recruited to those regions or are not available at this stage. In a sense the chromatin could be primed for gene expression changes later in development. Indeed, when we assessed whether these genes were affected at 5 dpf, of the positive ATAC genes, 3 genes (*bdnf*, *igfbp1a* and *vax2*) showed an increase in gene expression following the short exposure up to 50% epiboly and the recovery period to 5 dpf.

From our data, it remains unclear how Ezh2 is exactly involved in increased lipid accumulation. Gene expression profiles of TBT and the longer Ezh2i exposure showed a large overlap. Although only a relatively small set of genes were analyzed, these results might indicate that TBT acts via a similar mechanism as shown in mammalian cell models. Activation of RXR by TBT or RXR agonists in committed preadipocytes resulted in lower expression of *Ezh2* leading to overall lower levels of H3K27me3 and increased lipid accumulation [20]. If we extrapolate this to our findings in zebrafish, we see a decreased expression of *rxraa* following Ezh inhibition, which might be a negative feedback towards RXR, to prevent further lipid accumulation. Still, detailed mechanistic research is needed to address the exact mechanisms of action.

The question remains if we can use chromatin status as an endpoint in toxicological research and if this can be linked to differentially expressed genes. We have shown that no change in the accessibility at the *cebpa* locus does not mean there is no change in H3K27me3 or gene expression as *cebpa* showed a strong decrease in H3K27me3. This was reflected in lower gene expression levels at 5 dpf. The mechanisms behind this regulation are currently unclear and are subject for further investigation, but it does show that ATAC-seq data in itself might not give a complete view on the epigenetic status. Conversely, a change in chromatin accessibility is not always linked to gene expression. Cross-talk between epigenetic marks such as DNA methylation and histone PTMs and recruitment of transcription factors will in the end determine the expression of a gene [46]. Therefore, we think that multiple endpoints for epigenetic modifications should be assessed before drawing any conclusion about changes in epigenetic status. Nevertheless, the structural analysis of chromatin such as ATAC-seq seems a reasonable starting point for addressing changes at underlying epigenetic marks.

## Conclusions

Here, we show for the first time the applicability of ATAC sequencing in a toxicological setting in zebrafish following Ezh2 inhibition. Ezh2i increased lipid accumulation in the same manner as the positive control TBT, and ATAC profiles revealed involvement in metabolic processes and lipid metabolism. Gene expression profiles showed similar responses between TBT and Ezh2i. Our data further implies that Ezh2 inhibition in zebrafish might have a similar outcome for increased lipid accumulation as in mammals, possibly via conserved pathways, but further research into this is needed. Although our data does show that underlying epigenetic marks might still be important to assess as well, ATAC sequencing holds promise for toxicological purposes in the future.

## Methods

### Zebrafish husbandry

At Utrecht University, wild-type zebrafish (AB) were maintained in tanks in a continuous flow system ZebTec (Techniplast). Tanks were filled with reverse osmosis water and 15% daily water exchange, kept at 28 °C and photoperiod of 10 h dark and 14 h light. Zebrafish were fed two times a day with Gemma Micron 300 (Skretting) and once with live brine shrimp. Male and female fish (1:1 ratio) were separated the night before mating and placed in the same tank with a divider separating them. The following morning, the divider was removed, and embryos were collected and transferred to petri dishes containing E3 embryo medium (5 mM NaCl, 0.17 mM KCl, 0.33 mM CaCl_2_∙2H_2_O, 0.33 mM MgSO_4_∙7H2O). At the day of exposure, fertilized eggs were selected for exposure experiment. Our experimental design did not need ethical committee approval as embryos used were beyond the stage defined as an animal experiment (≤ 5dpf). Our fish stocks are housed under license AVD 1080020197366.

The Norwegian University of Life Sciences (NMBU) zebrafish facility is licensed by the Norwegian food inspection authority (NFIA) permit no. 5793. AB wild-type zebrafish were maintained according to standard operating procedures with 28 °C and photoperiod of 10 h dark and 14 h light as described earlier [47].

### Developmental exposure to PF-06726304 acetate (Ezh2i)

PF-06726304 acetate (5,8-dichloro-2-[(1,2-dihydro-4,6-dimethyl-2-oxo-3-pyridinyl)methyl]-7-(3,5-dimethyl-4-isoxazolyl)-3,4-dihydro-1(2*H*)-isoquinolinone acetate, CAS Number 1616287-82-1, 99% purity) was purchased from Sigma-Aldrich, and dissolved in dimethyl sulfoxide (DMSO, Sigma-Aldrich, D8418, purity > 99,9%) to obtain a stock concentration of 50 mM. Other dilutions were prepared from this stock (10 mM, 1 mM, 0.1 mM, and 0.01 mM) in order to have a final DMSO concentration of 0.1 and 0.01% during exposure to zebrafish embryos. A 24-well plate was set up with control and test concentrations ranging between 0.01 and 50 μM, each with four replicates. On average, 10 embryos, all in the same developmental stage (4–8-cell stage), were placed in each well and incubated at 26 °C. Defects on development at 1 dpf, 2 dpf and 5 dpf were observed under a Motic stereo microscope (Motic SMZ-171T) and recorded on score sheets and percentage of mortality and percentage of effected embryos were calculated. Using GraphPad (V8) the EC50 and LC50 at 5 dpf was calculated. After screening, each well was photographed using the camera (Moticam 5, 5.0MP (2592 × 1944), CMOS sensor) of the stereomicroscope with (10× 2× 0.5×) 10 times magnification using manufacturer’s software (Motic Images Plus 3.0). Images were captured in TIFF format.

For lipid staining and qPCR analysis we used 6-well plates with each four replicates per chemical exposure. Per well we exposed 50 embryos in 5 mL exposure medium containing 0.01% DMSO (solvent control), positive control 1 nM TBT, short exposure to Ezh2i up to 50% epiboly which was refreshed with solvent control 0.01% DMSO, and long exposure to Ezh2i up to 5 dpf. Two independent experiments were performed. Per well, 10 embryos were fixated for lipid staining, and embryos were snap frozen in liquid nitrogen for qPCR analysis.

For omics analysis, embryos were collected directly after fertilization and kept at 28 ± 1 °C. Non-viable embryos were discarded from the exposures. Chemical exposure to the Ezh2i was performed in a 6-well plate, and each exposure was performed in triplicate. For ATAC-seq, 150 embryos were maintained in 9 mL of 5 μM PF-06726304 acetate and 150 embryos were maintained in 9 mL control solution (0.05% DMSO). The exposure time was from approximately 2-cell stage to 50% epiboly. For ChIP-qPCR, 200 embryos were maintained in 9 mL of 5 μM PF-06726304 acetate and 200 embryos were kept in 9 ml solvent control solution (0.05% DMSO).

### Oil Red O staining

For the whole mount Oil Red O (ORO) staining, 5-dpf-old embryos were fixated in 4% paraformaldehyde overnight at 4 °C. After a short wash with PBST (PBSzero with 0.05% Tween 20), three washes (3 × 10 min) with only PBSzero were performed. Next, we incubated the embryos in 60% isopropanol for 1 h, followed by a 0.3% ORO staining in 60% isopropanol for 75 min. After the staining, the embryos were immediately transferred in PBST (PBSzero with 0.1% Tween 20). ORO stained embryos were transferred to 3% methylcellulose in order to image them using a Zeiss Stemi SV11 microscope and a Nikon DXM1200 Digital Camera. The images were acquired using the Nikon ACT software (vs 2.63).

### Measuring area of lipid staining

In the original files, four or five 5-dpf larvae were included per image. In order to avoid any measurement error, we made individual images by extracting the single larvae using Adobe Photoshop, without any further image manipulation. For each chemical exposure (DMSO, TBT, Ezh2i 50%epiboly and Ezh2i 5 dpf), we measured Oil Red O staining in the abdominal region of larvae using Image J software (https://imagej.nih.gov/ij/) with the following settings (*Image *> *Adjust *> *Color Threshold*) Hue = 0; Saturation levels set at values between Brightness depending on the histogram shown per image, followed by Threshold color (Red) and Color Space (HSB). Then the images were filtered by red pixels and measured for the numbers of pixels containing a certain threshold for the red color. (*Analyze *>* Measure*). Per exposure we calculated the average area stained by Oil Red O.

### Assay for transposase-accessible chromatin

The chorion was removed with pronase (0.3 mg/mL pronase) and the yolk was dissociated in 500 μL Ginzburg fish Ringer deyolk buffer (55 mM NaCl, 1.8 mM KCl, 1.25 mM NaHCO_3_) and the cell membrane was lysed in 50 μL lysis buffer (10 mM Tris–HCl pH 7.4, 10 mM NaCl, 3 mM MgCl_2_, 0.1% Igepal CA630). A 2X DNA tagmentation buffer (22 mM Tris–HCl pH 7.4, 10 mM MgCl_2_, 20% Dimethylformamide; pH 7.4) was prepared. The isolated nuclei were resuspended in 50 μL transposition reaction mixture (25 μL 2X DNA tagmentation buffer, 1.25 μL Tn5 (Illumina, 15027865), 23.75 μL H_2_O). The samples were incubated on Thermomixer at 300 rpm, 37 °C for 30 min. The tagmented DNA was purified with the MinElute PCR Purification Kit (QIAGEN, cat. #28004) and eluted in 10 μL elution buffer. The Nextera Index Kit (Illumina, FC-121-1030) was used for the library preparation. Real-time PCR was performed using 1 μL of the transposed DNA and 9 μL qPCR master mix (1X NEB Next High-Fidelity PCR Master Mix (New England Labs, cat. #M0541), 1X SybrGreen, 0.25 nM indexed primers) to determine the number of PCR cycles needed for the library preparation. The temperature regime for the thermocycling was (1) 72 °C for 5 min, (2) 98 °C for 30 s, (3) 98 °C for 10 s, (4) 63 °C for 30 s, and (5) 72 °C for 30 s. Step 3–5 was repeated 20 times. The preferred number of amplification cycles for the libraries was decided to be 9–10 cycles. The remaining 9 μL of purified transposed DNA was PCR amplified with 41 μL PCR master mix (1X NEB Next High-Fidelity PCR Mastermix (New England Labs, cat #M0541), 5 μM common and specific index primers (Nextera Index Kit, Illumina). The temperature regime for the thermocycling was (1) 72 °C for 5 min, (2) 98 °C for 30 s, (3) 98 °C for 10 s, (4) 63 °C for 30 s, and (5) 72 °C for 60 s. The amplified library was purified using the MinElute PCR Purification Kit and eluted in 20 μL of elution buffer. The libraries were cleaned up on a 2% agarose gel (Xe dry gel system, Invitrogen). During blue light excitation, the < 100 bp and > 15 kb localizations were removed, and the DNA libraries of interest were extruded from the gel using Invitrogen PureLink Quick Gel Extraction Kit (Invitrogen, cat. #K2100). The libraries were pair-end sequenced with a read length of 150 bases and a read depth of 50 million raw reads. The sequencing was outsourced to Novogene, Hong Kong, China.

### Raw data processing and statistical analysis

Raw data files were mapped to GRCz11 using the bioinformatics pipeline snakePipes using the ATAC seq mode [48]. In this pipeline, fastq files are quality and adapter trimmed with Trim galore!, aligned with Bowtie2 and peaks are detected with MACS2. Due to too low read depths we needed to adjust the analysis by including 1 nucleosome by increasing the fragment length to 300 bp. The obtained MACS peaks from each replicate were merged, and overlapping peaks were merged to one peak, resulting in 65336 peaks. The genomic locations were imported in Seqmonk v.1.45.4 and analyzed using EdgeR on raw read counts, setting the FDR at 0.05.

For Fig. 3e, published data sets were used to associate the observed ATAC peaks to different histone post-translational modification and another ATAC dataset in zebrafish, namely ATAC-seq (PRJCA000283) [34], histone H3 lysine 4 trimethylation (H3K4me3), H3K4me1, H3K27ac (GSE:32483) [35], H3K27me3 and H3K36me3 (GSE44269) [36] data from dome stage (4.3 hpf). After adapter and quality trimming with Trim galore! (v 0.4.1, Babraham Institute, UK), Bowtie2 (v2.2.9) was used for mapping. Bam files were loaded in Seqmonk (v1.45.4) and log2 normalized read counts were measured 10 kb up and downstream of ATAC peaks.

### Chromatin immunoprecipitation

Chromatin immunoprecipitation (ChIP) was done as described in Lindeman et al. [49]. Antibodies, H3K4me3 (cat #. C15410003), H3K27me3 (cat # C15410069) and H3-pan (cat# C15310135) were purchased from Diagenode, Belgium. ChIP-primers for *cebpa* locus were designed in Primer3 v4.0.0 [50, 51] and purchased from ThermoFisher Scientific (Additional file 1: Table S1). The precipitated DNA was quantified with qPCR (SYBR green, Roche) using 2.5 µL ChIP DNA as input template. Both ChIP and the qPCR experiments were performed in duplicates.

### DATA analysis

Genes associated with significant ATAC peaks at their TSSs(349 genes) were used for gene ontology (GO) analysis (Gene Set Enrichment Analysis, gene ontology for biological processes) using the WebGestalt (WEB-based GEne SeT AnaLysis Toolkit) online tool [52, 53]. The complete list of ATAC peaks located at TSSs was used as background for the overrepresentation analysis.

As a second dataset we selected all DEGs from 3.3 hpf *MZEzh2* mutant vs wild-type embryos [37] with an adjusted p-value < 0.05 (2904 genes). Using a Venn plot diagram (http://bioinformatics.psb.ugent.be/webtools/Venn/) we compared this dataset with the gene list after ATAC-seq and the overlapping genes were subjected to GO term analysis as well.

### RNA extraction and cDNA synthesis

Per experimental group (DMSO 0.01%; 1 nM TBT, short and long exposure to 5 µM Ezh2i), we had four replicates using 50 pooled embryos at 50% epiboly and 10 embryos at 5 dpf. Samples were taken from two independent exposure experiments, and snapfrozen and kept at − 80 °C Tissue homogenization was achieved using the Qiagen Tissue homogenizer: 2 × 30 s at 6000 rpm, and RNA extraction was performed using the NucleoSpin RNA Plus purification kit (Macherey-Nagel GmbH & Co. KG, Düren, Germany, Cat. No. 740984.50). After RNA extraction, an extra DNase I treatment was performed to remove genomic DNA. Per sample we added 4 µL 10× buffer; 1U/sample DNaseI RNase free (Roche, 4716728001). The tubes were incubated for 15 min at 37 °C after which we added 1/10 vol 3 M NaAc, 2 vol of ice-cold 100% ethanol (Merck, purity > 99.2%), 1 µL of GlycoBlue (Ambion, AM9516) and precipitated overnight at − 20 °C. The tubes were centrifuged at 13.000 rpm for 30 min, and the precipitate was washed with 70% ethanol. Tubes were centrifuged for 15 min at 13.000 rpm. Supernatant was removed, and pellet was air-dried for 10 min. RNA was dissolved in 30 µL UltraPure DEPC water (Invitrogen, 750024)

Total RNA concentration was measured using a NanoDrop 2000 (ThermoFisher) spectrophotometer. One µg of RNA was used for first-strand cDNA synthesis using the High-Capacity cDNA synthesis kit (Thermo Fischer, Applied Biosystems™, Foster City, California, USA, Cat. No. 4368814), and contained 8 mM dNTPs, random hexamers, 5 U/µL reverse transcriptase and 1.0 µg total RNA in a final volume of 20 µL. Reaction conditions were: 10 min 25 °C, 120 min 37 °C and 5 min 85 °C. After synthesis, 180 µL of DEPC treated water is added to a final volume of 200 µL (Invitrogen, Part no: 46-2224).

### Gene expression analysis

We have selected genes that are known to have a role in the adipose tissue–gut–brain pathway and lipid metabolism and four reference genes (*hmbs, gapdh, hprt1, nono*) were added to normalize our datasets. Next to that, we performed qPCR analysis for genes that did show a changed chromatin structure after ATAC-seq. Primers were developed using the primer blast tool from NCBI (https://www.ncbi.nlm.nih.gov/tools/primer-blast/), with an amplicon length between 70 and 200 bp, and annealing temperatures of 60 degrees, intron spanning, preferably one primer overlapping an exon–exon junction. For every primer set the efficiency was determined using serial dilutions of cDNA, and checked by gel electrophoresis. Table with primer sequences for target genes is available in Additional file 1: Table S3. QPCRs reactions were performed as described in den Broeder et al. [23].

### Statistical analysis

Principal component analysis was performed using the online ClustVis tool (https://biit.cs.ut.ee/clustvis/) [54]. As input we have used the individual replicated qPCR expression values per sample per exposure condition.

Change in lipid accumulation in 5-dpf-old larvae was analyzed by one way ANOVA analysis using Graphpad Prism 8.

The Cq determination was done by regression and extracted from BioRad CFX Maestro software. Normalized Δ∆*C*q levels were calculated, and significance analysis was performed on the log2 transformed values that were obtained via two-way ANOVA analysis with a Dunnett’s multiple comparison post hoc analysis in GraphPad Prism 8. Results are presented in a heatmap at 5 dpf (GraphPad Prism 8) using the fold change expression values.

ChIP results were analyzed by two-way ANOVA with Sidak multiple comparisons correction using Graphpad Prism 8.

## Supplementary information

**Additional file 1: Figure S1.** Phred scores after sequencing analysis. **Figure S2.** Mapping analysis of data after alignment to the genome. **Table S1.** Primer sequences used for ChIP analysis at *cebpa* gene locus. **Table S2.** Checklist for QPCR according MIQE guidelines. **Table S3.** Gene ID and sequences used for qPCR.

**Additional file 2.** A Microsoft Excel spreadsheet that contains Gene Ontology terms for **Tab 1**. Biological function **Tab 2.** cellular function, and **Tab 3.** molecular function.

## Data Availability

The data discussed in this publication have been deposited in NCBI’s Gene Expression Omnibuss [55], and are accessible through GEO Series accession number GSE140233 (https://www.ncbi.nlm.nih.gov/geo(query/acc.cgi?acc=GSE140233).

## Abbreviations

ATAC: Assay for transposase-accessible chromatin
BPA: Bisphenol A
DEG: Differential expressed genes
DEP: Differential enhanced peaks
dpf: Days post-fertilization
Ezh: Enhancer of zeste
hpf: Hours post-fertilization
GO: Gene ontology
MBT: Mid-blastula transition
MDCs: Metabolism disrupting chemicals
NAFLD: Non-alcoholic fatty liver disease
PRC2: Polycomb repressive complex 2
PTMs: Post-translational modifications
ROSI: Rosiglitazone
T2DM: Type 2 diabetes mellitus
TBT: Tributyltin
ZGA: Zygotic genome activation
