# Supplementary material for: Inhibition of methyltransferase activity of enhancer of zeste 2 leads to enhanced lipid accumulation and altered chromatin status in zebrafish
Source: Epigenetics Chromatin. 2020 Feb 12;13:5. doi: 10.1186/s13072-020-0329-y (PMC7014624; doi:10.1186/s13072-020-0329-y)
Supplement: Supplementary file 1 — Additional file 1: Figure S1. Phred scores after sequencing analysis. Figure S2. Mapping analysis of data after alignment to the genome. Table S1. Primer sequences used for ChIP analysis at cebpa gene locus. Table S2. Checklist for QPCR according MIQE guidelines. Table S3. Gene ID and sequences used for qPCR. [file 13072_2020_329_MOESM1_ESM.docx]

Inhibition of Methyltransferase Activity of Enhancer of Zeste 2 Leads to Enhanced Lipid Accumulation and Altered Chromatin Status in Zebrafish

Marjo J. den Broeder ^1^, Jarle Ballangby ^2^, Leonie M. Kamminga ^3,4^, Peter Aleström ^2^, Juliette Legler ^1,5^, Leif C. Lindeman ^2^, Jorke H. Kamstra ^1,2^

1. Institute for Risk Assessment Sciences, Faculty of Veterinary Medicine, Utrecht University, Utrecht, The Netherlands
2. Faculty of Veterinary Medicine, Norwegian University of Life Sciences, Oslo, Norway
3. Radboud University Nijmegen, Faculty of Science, Department of Molecular Biology, Radboud Institute for Molecular Life Sciences, Nijmegen, The Netherlands
4. Wageningen University and Research Library, Wageningen , The Netherlands.
5. Utrecht Institute for Pharmaceutical Sciences, Faculty of Science, Utrecht University, The Netherlands

Additional figures and tables

Figure S1: Phred scores after sequencing analysis

Figure S2: Mapping analysis of data after alignment to the genome.

Table S1: Primer sequences used for ChIP analysis at *cebpa* gene locus

| **Gene** | **Start** | **End** | **Forward primer** | **Reverse primer Temp.** | **(°C)** |
| --- | --- | --- | --- | --- | --- |
| cebpa | -4332 | -4200 | TCTCTGGCGCAACTTCCAAT | TCTGGGTCGAACAAATGGGT | 60 |
| cebpa | -902 | -767 | CATTCTTTAGGTGCCAGGCC | CCCCATAGTGCGAGAAAAGC | 60 |
| cebpa | -123 | 21 | TAGGTCTATCAGTGCGTCCG | ACTTGCAACCTCAGTGTGTG | 60 |
| cebpa | 498 | 603 | GATGTATGGCTGCCTGAACG | TCCCGAGGCTCTTGTTTGAT | 60 |
| cebpa | 1840 | 1943 | GCAGTGAAGTCCTGTCTTGC | CACTGAAACCATCTCTGCTCG | 60 |

Table S2: Checklist for QPCR according MIQE Guidelines

| **Sample preparation** |  |
| --- | --- |
| 1. Fresh - How rapidly processed? | No |
| 1. Frozen - How frozen? | Snapfrozen at 5 dpf |
| 1. Whole vs. microdissected | Whole embryo |
| 1. Sample storage conditions and duration | -80 ̊C, two weeks |
| 1. Fixed - How fixed, how old? | Not fixed |
| **Nucleic Acid** |  |
| 1. Quantification | Total RNA concentration was measured using a Nanodrop 2000 (ThermoFisher) spectrophotometer |
| 1. Quality/integrity | Quality of RNA was checked by Nanodrop 2000 |
| 1. Inhibition dilution or spike | Not Done |
| 1. DNA contamination assessment of RNA sample | Assessing DNA contamination using NanoDrop 2000. |
| 1. DNase treatment | Extra DNAse I treatment after RNA extraction is standard performed. Per sample we added 4 µL 10x Buffer; 1U/sample DNaseI RNase free (Roche, 4716728001). The tubes were incubated for 15 min at 37°C The tubes were incubated for 15 min at 37°C after which we added 1/10 vol 3M NaAc, 2 vol of ice-cold 100% ethanol (Merck, purity> 99,2%), 1 µL of GlycoBlue (Ambion, AM9516) and precipitated overnight at -20°C. The tubes were centrifuged at 13.000 rpm for 30 min, and the precipitate was washed with 70 % ethanol. Tubes were centrifuged for 15 min at 13.000 rpm. Supernatant was removed, and pellet was air-dried for 10 min. RNA was dissolved in 30 µL UltraPure DEPC water (Invitrogen,750024) |
| 1. Manufacturer of reagents used | RNA extraction was performed using the NucleoSpin RNA Plus purification kit (Machery-Nagel GmbH & Co. KG, Düren, Germany, Cat. No. 740984.50) |
| 1. Amount of sample used for extraction | 50 embryos at 50% epiboly  10 larvae at 5 dpf |
| **Reverse transcriptation** |  |
| 1. cDNA priming method + concentration | First-strand cDNA synthesis using the High-Capacity cDNA synthesis kit (Thermo Fischer, Applied Biosystems^TM^,Foster City, California, USA, Cat. No. 4368814), and contained 8 mM dNTPs, random hexamers, 5 U/µL reverse transcriptase. . After synthesis, 180 µL of DEPC treated water is added to a final volume of 200 µL (Invitrogen, Part no: 46-2224). |
| 1. Amount of RNA used per reaction | 1 µg |
| 1. Enzyme type and concentration | MultiScribe® Reverse Transcriptase (50 U/µL) |
| 1. Detailed reaction conditions | 10 min 25 °C, 120 min 37 °C and 5 min 85 °C |
| 1. Manufacturer of reagents used | Thermo Fischer, Applied Biosystems^TM^,Foster City, California, USA, Cat. No. 4368814 |
| 1. Reaction volume | 20 µl |
| 1. Storage of cDNA | Short -20 °C; long term -80°C |
| **Gene Target** |  |
| 1. Database name and target gene accession number | All needed information for this section is provided in Supplemental Table 3 |
| 1. Intronless, targeting of all splice variants/splice variant-specific targeting | Annealing temperatures of 60 degrees, intron spanning, preferably one primer overlapping an exon-exon junction. |
| 1. Official gene symbol | According Zebrafish Nomenclature used on ZFIN |
| 1. Information about (retro)pseudogenes | Not Done |
| **Primers** |  |
| 1. Primer sequences | All needed information for this section is provided in Supplemental Table 3 |
| 1. End concentration of primers used in assay | 250 nM |
| 1. Primer purification method | Desalted |
| 1. Manufacturer of oligonucleotides | Sigma-Aldrich |
| **Assay details** |  |
| 1. Amplicon length | 70-200 bp |
| 1. Specific BLAST or equivalent in silico specific screen | https://www.ncbi.nlm.nih.gov/tools/primer-blast/ |
| 1. Experimental validation of specificity | All PCR products are checked by gel-electroforesis |
| 1. NTC; Sensitivity | Cq values should be <30. Otherwise excluded from analysis |
| 1. PCR efficiency, PCR efficiency standard curve slope and r-squared value | For every primer set the efficiency was determined using serial dilutions of cDNA, r-squared value was determined. Depending on time of expression this was done using cDNA extracted from embryos at 50% epiboly or at 5 dpf |
| 1. RTPrimerDB ID | Not Applicable |
| **PCR Cycling** |  |
| 1. Amount of cDNA/DNA used per reaction | 1 µL from the diluted cDNA (200 µL) |
| 1. Detailed reaction conditions, thermocycling parameters | QPCR reaction conditions were: 15 min 95 °C followed by 40 cycles of 15 s 95 °C, 45 s 60 °C and plate analysis |
| 1. Manufacturer of reagents used | SYBR Green universal supermix (Bio-Rad, 172-5121) |
| 1. Manual/robotic dispensing of reagents | Multi D pipet from Biohit |
| 1. Manufacturer of plates/tubes | Hard Shell PCR plate 96-well (HSP9631, Bio-Rad)  Microseal B seal (MSB1001, Bio-Rad) |
| 1. Manufacturer of real-time instrument | CFX96 Real Time System, Bio-Rad |
| **Data analysis** |  |
| 1. Cq value determination method | The Cq determination was done by regression. Cq values should be <30 |
| 1. Treatment of NTCs and technical replicates | No technical replicate |
| 1. Normalisation method | The Cq determination was done by regression, and the fold change in expression (Δ∆Cq) was calculated using the BioRad Maestro analysis software. Significance analysis was performed on the log2 transformed values that were obtained via ANOVA analysis. Hierarchical clustering of log2 normalized fold changes was performed in GraphPad Prism 8 using the fold change expression values. |
| 1. Is r-squared value of regression curve satisfactory? | Regression curve values |
| 1. Has assay sensitivity been adequately evaluated and described? | Sensitivity of target genes at different embryonic stages have been investigated |
| 1. Is the dynamic range of the assay acceptable? | Cq values between 20-30 |
| 1. Is the coefficient of variation for inter and intra-assay reproducibility reasonable? | On every plate, inter-run calibration samples were included as well |
| 1. Concordance of biological replicates | 4 biological replicates |
| 1. Analysis program | BioRad CFX Maestro software, GraphPad |
| 1. Assay carried out by core lab or investigator's lab | Investigator's lab |
| 1. Acknowledgement of author's contribution to analysis and interpretation | Done |
| 1. Submission of Cq values of raw data using RDML | At this moment raw data is available upon request. We will make all raw data public available. |

Table S3: Gene ID and sequences used for qPCR

| **ENSDARG** | **Accession number** | **Target gene** | **Oligo sequence forward** | **Oligo sequence reverse** |
| --- | --- | --- | --- | --- |
| ENSDARG00000040298 | NM_001079861.1 | apoa4b | AACTGCAGAGGAAACCGTCA | GCTCCCTCAGCACTTCAGTC |
| ENSDARG00000067976 | NM_001083123.1 | ar | CTCCGCACGAGCAGTGGTA | GTCAAACCTGCCATCCGTGAA |
| ENSDARG00000018817 | NM_131595.2 | bdnf | GGACACTTTCGAGCAGGTCA | CTCCAAAGGCACTTGGTTGC |
| ENSDARG00000036074 | NM_131885.2 | cebpa | GGAGCAAGCAAACCTCTACG | GATCTCGCTCAGGTCTCCAG |
| ENSDARG00000020850 | NM_131263 | ef1a | TTGAGAAGAAAATCGGTGGTGCTG | GGAACGGTGTGATTGAGGGAAATTC |
| ENSDARG00000037894 | NM_001039983 | ezh1 | AGGAAGCGTCTAGTGAGGTCT | ACGGCGATTTGACTGGAACA |
| ENSDARG00000010571 | NM_001077279.1 | ezh2 | AAATCGGAGAAGGGTCCTGT | TCTGTTGGAGCTGAACATGC |
| ENSDARG00000019357 | NM_001044712 | fabp1a | ACACTGGTCAACACTCTGACG | AAGTCTTGCGTGTGTTTGCTC |
| ENSDARG00000043457 | NM_001115114 | gapdh | CGACTCCACCCATGGAAAGT | TAATGTTGGCTGGGTCCCTC |
| ENSDARG00000008840 | NM_201154 | hmbs(a) | GTGTGTGGAATTGGACAACAAAGTG | CGAGGGCTGATGATGAGATATTGC |
| ENSDARG00000008884 | NM_212986 | hprt1 | CAGCGATGAGGAGCAAGGTTATG | GTCCATGATGAGCCCGTGAGG |
| ENSDARG00000059280 | NM_131125.1 | hoxd3a | GAAAGAGACCCGCCAAAATGC | GTCATCGCAGGTTTCTCCTGCT |
| ENSDARG00000059274 | NM_131126.3 | hoxd9a | ACAACAACTTGACCCAAGCAAC | ATTTGGTGTACGGACAACGCT |
| ENSDARG00000099351 | NM_173283.3 | igfbp1a | AGGCCAAAGTCAACGCGATA | TTGTCAAGGGCTGTCTGGAG |
| ENSDARG00000025348 | NM_001126463.1 | igfbp5b | ACCCACCCATTGATCGTGAA | AGCCTGCTTCTTGCTGTTGA |
| ENSDARG00000011948 | NM_001142672.1 | insra | GATTCAGATGGCCGCAGAGA | AGACCCTTCCCACCCTTTCT |
| ENSDARG00000071524 | NM_001123229.1 | insrb | GGGCAGGGATCATTTGGGAT | CCATGACAACCAATGTCGGC |
| ENSDARG00000087697 | NM_131127.1 | lpl | GGCCAAATTTGTCAACTGGT | CATGAGCGCCAAGACTGTAA |
| ENSDARG00000020482 | NM_201579 | nono | ATGGACAACACACCGATGCT | AAATCTCCGAAGCCTTGCCA |
| ENSDARG00000098439 | NM_001017545 | nr1h3 (lxra) | AGACCAGATCGCCCTACTCA | GCTGTCAATCGCTGGGTTAT |
| ENSDARG00000052695 | NM_131180.1 | nr2f1a | TTTAAAAGTGGGCATGCGGC | GACGTAGGATAAGGCTCGGC |
| ENSDARG00000031777 | NM_001161333.1 | pparaa | CGACGACACCTTCAACTCCT | GAGATCCGGATGAGTTCTCG |
| ENSDARG00000054323 | NM_001102567.1 | pparab | GTGACCTCGCACTGTTTGTC | CTCCTGCATTCGCTCGATA |
| ENSDARG00000044525 | XM_005168286.2 | pparda | ATCGACCTCTTCCTCAACGA | AAGCAGCCCGTCTTTATTCA |
| ENSDARG00000009473 | NM_131468.2 | ppardb | CGTCAACACAGCCTACCTGA | GCCACAGGGAGTCCATATCA |
| ENSDARG00000031848 | NM_131467.1 | pparg | TACGAGAACAACCCAACA | GTATCTGCTGTGCTCTGTA |
| ENSDARG00000057737 | NM_001161551 | rxraa | ACCCCACTCTCTCTCACCAA | GCAGCTGTACACACCGTAGT |
| ENSDARG00000012667 | XM_005160883.4 | tfap2b | GTTTTTGCACCGTGTGGTCA | TGGCGATCCGTGGAGGAATA |
| ENSDARG00000027087 | NM_194385.1 | tgfb2 | ACGCGCTTTGCAGGTATAGA | AATGTGGAGACTGACCGCTG |
| ENSDARG00000058702 | NM_194409.1 | vax2 | CAGAGCTCTACCGACACACC | ATAGTGCCCTTGGCGTCTCT |
| ENSDARG00000032234 | NM_001044344.1 | wnt2bb | CGAACTGTGAAAGACGCACG | GCGCTTTACAGCCATTCTTCC |
| ENSDARG00000040925 | NM_178219.2 | wnt10b | CCGGATGAGGTCACAATGCT | TGGCACTTGCACTTTCTCCT |
| ENSDARG00000102464 | NM_130937.1 | wnt5b | CCCTCATCGTCTGCAACTCA | TCTGACCCTGAGATAGGCCC |
